# Never say never: a novel approach to tackle a bleeding ectopic varix

**DOI:** 10.1055/a-2408-9639

**Published:** 2024-09-19

**Authors:** Akshay Kulkarni, Anup Nair, Siddhant Mehta, Bhushan Bhaware, Saurabh Mukewar, Shrikant Mukewar

**Affiliations:** 1 Gastroenterology, Midas Multi-speciality Hospital, Nagpur, India

A 53-year-old woman with decompensated cirrhosis due to nonalcoholic steatohepatitis presented with hematochezia. An upper gastrointestinal endoscopy revealed small varices that were not amenable to endoscopic variceal ligation. A computed tomography (CT) scan with intravenous contrast revealed multiple large collaterals close to the terminal ileum ( Fig. 1 ; Video 1 ), but with no sign of portal or mesenteric thrombosis. The patient had continuous hematochezia with transfusion-dependent anemia. Colonoscopy revealed a large amount of fresh and altered blood in the terminal ileum ( Fig. 2 ) and the patient continued to have persistent bleeding, requiring transfusions and vasopressors. Angiography revealed no evidence of arterial bleeding ( Fig. 3 ); portal vein access with venogram was avoided owing to her ascites and thrombocytopenia. The patient’s relatives declined surgery on her behalf owing to the possibility of further decompensation, and therefore rectal endoscopic ultrasound (EUS) was planned.

**Fig. 1:**
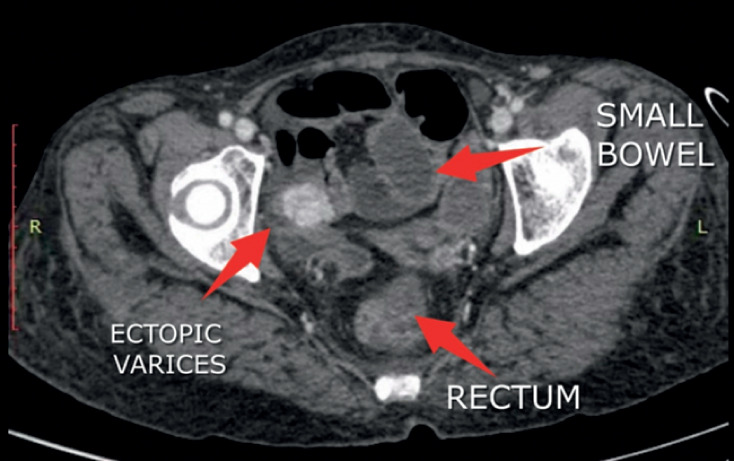
Contrast-enhanced computed tomography image showing a large group of collaterals in close proximity to the terminal ileum.

**Fig. 2:**
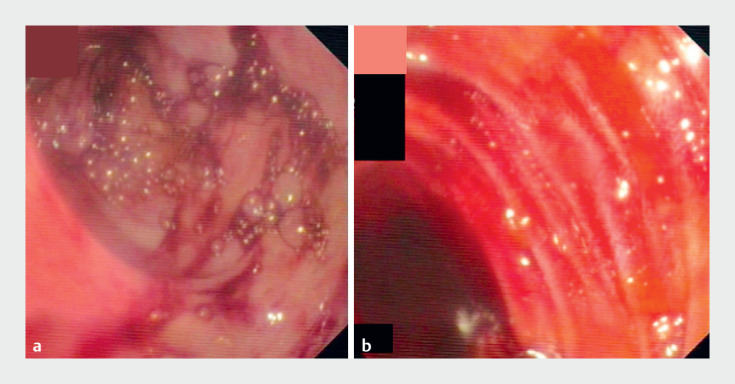
Colonoscopic images showing fresh blood in the: **a** colon; **b** terminal ileum, suggesting a small-bowel bleed.

**Fig. 3:**
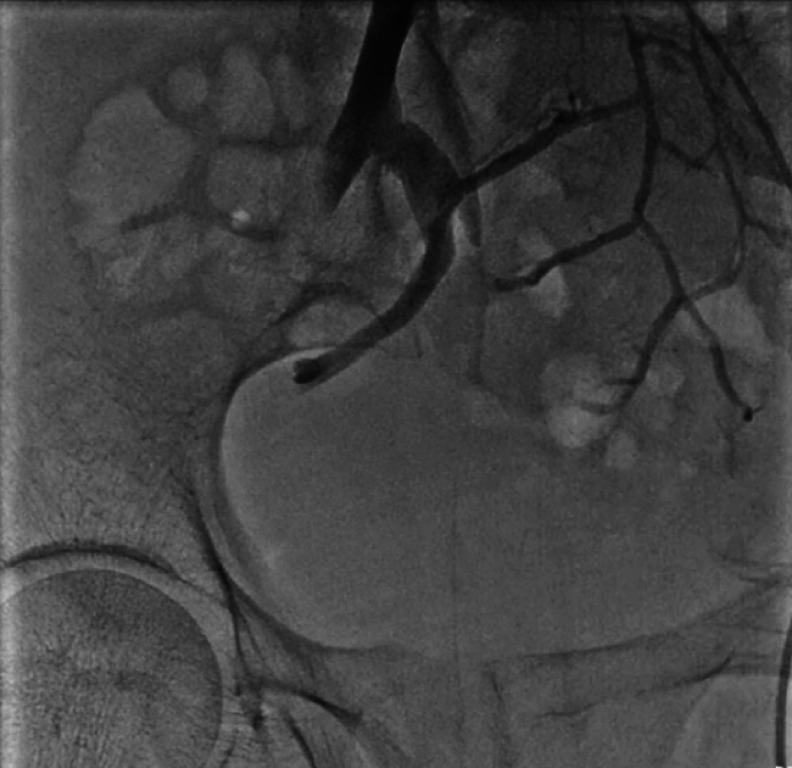
Digital subtraction angiography image showing no evidence of arterial bleeding, with direct portal vein puncture or cannulation via a transjugular intrahepatic portosystemic shunt avoided owing to the patient’s thrombocytopenia.

Ileal varices are seen on contrast-enhanced computed tomography and rectal endoscopic ultrasound (EUS), which also showed significant ascites. After paracentesis had been performed, coil insertion and glue injection were performed under EUS guidance, with subsequent Doppler examination showing obliteration of the varices.Video 1

The rectal EUS ( Fig. 4 ) was performed with the ME-3 scope (Olympus, Japan). It showed significant ascites and large juxta-ileal varices. After paracentesis had been performed, the EUS was repeated and a 19G needle (Boston Scientific, USA) was passed into the varices. After saline had been injected, a 15-mm metal coil (Nester; Cook Medical, USA) was passed into the varix, followed by 3 mL of glue (Endocryl, India) and a saline flush. Doppler ultrasound showed obliteration of the flow in the varix. The patient recovered uneventfully and was discharged in a stable condition.

**Fig. 4:**
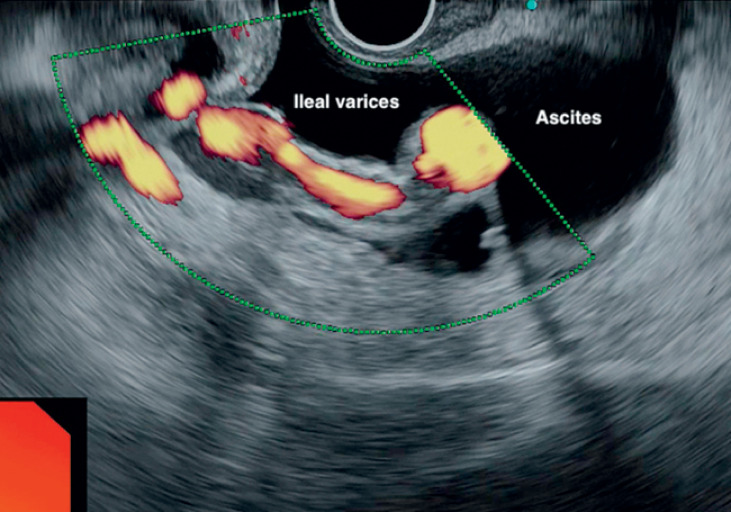
Rectal endoscopic ultrasound image showing significant ascites with ileal varices floating in the fluid.

Ileal varices have been documented in a considerable proportion of cirrhotic patients and bleeding from these varices is not rare 1 . Such bleeding has previously been managed either with multiple radiologic interventions 2 3 or with surgical resection and anastomosis 4 . To the best of our knowledge, this is the first case describing transrectal EUS-guided coiling of ileal ectopic varices. This procedure may be safe, feasible, and effective in selected cases, where the anatomy allows direct transmural access to the collaterals without the presence of intervening structures.

Endoscopy_UCTN_Code_TTT_1AS_2AG

## Notes

Endoscopy E-Videos https://eref.thieme.de/e-videos
*E-Videos* is an open access online section of the journal *Endoscopy* , reporting on interesting cases and new techniques in gastroenterological endoscopy. All papers include a high-quality video and are published with a Creative Commons CC-BY license. Endoscopy E-Videos qualify for HINARI discounts and waivers and eligibility is automatically checked during the submission process. We grant 100% waivers to articles whose corresponding authors are based in Group A countries and 50% waivers to those who are based in Group B countries as classified by Research4Life (see: https://www.research4life.org/access/eligibility/ ). This section has its own submission website at https://mc.manuscriptcentral.com/e-videos .

## References

[1] Misra SP Dwivedi M Misra V Ileal varices and portal hypertensive ileopathy in patients with cirrhosis and portal hypertension Gastrointest Endosc 2004 60 778 783 15557954 10.1016/s0016-5107(04)02049-8

[2] Hashimoto Y Amano H Fukumoto A Percutaneous transhepatic sclerotherapy for recurrent bleeding ileal varices diagnosed by capsule endoscopy and computed tomography during percutaneous transhepatic venography Hepatol Res 2013 43 436 440 23560865 10.1111/j.1872-034X.2012.01083.x

[3] Michielan A Vieceli F Pravadelli C Combination of transjugular intrahepatic portosystemic shunt and antegrade through-the-TIPS coil embolization for bleeding mixed-type ectopic ileal varices Clin J Gastroenterol 2023 16 668 672 10.1007/s12328-023-01830-w 37452994 PMC10539418

[4] Ueda J Yoshida H Mamada Y Successful emergency enterectomy for bleeding ileal varices in a patient with liver cirrhosis J Nippon Med Sch 2006 73 221 225 16936448 10.1272/jnms.73.221

